# Primary brain calcification in patients undergoing treatment with the biphosphanate alendronate

**DOI:** 10.1038/srep22961

**Published:** 2016-03-15

**Authors:** J. R. M Oliveira, M. F Oliveira

**Affiliations:** 1 Neuropsychiatric Department - Federal University of Pernambuco (UFPE), Recife, Pernambuco-Brazil; 2 Neurosurgery Department. Hospital do Servidor Público Estadual de São Paulo, São Paulo – SP, Brazil

## Abstract

Brain calcification might be associated with various metabolic, infectious or vascular conditions. Clinically, brain calcification can include symptons such as migraine, parkinsonism, psychosis or dementia. The term Primary Brain Calcification was recently used for those patients without an obvious cause (formerly idiopathic) while Primary Familial Brain Calcifications was left for the cases with autosomal dominant inheritance. Recent studies found mutations in four genes (*SLC20A2, PDGFRB, PDGFB* and *XPR1*). However, these gene represent only 60% of all familial cases suggesting other genes remain to be elucidated. Studies evaluating treatments for such a devastating disease are scattered, usually appearing as single case reports. In the present study, we describe a case series of 7 patients treated with Alendronate, a widely prescribed biphosphanate. We observed good tolerance and evidence of improvements and stability by some patients. No side effects were reported and no specific symptoms related to medication. Younger patients and one individual continuing a prescription (prior to study commencement) appeared to respond more positively with some referred improvements in symptoms. Biphosphanates may represent an excellent prospect for the treatment of brain calcifications due to their being well tolerated and easily available. Conversely, prospective and controlled studies should promptly address weaknesses found in the present analysis.

The term primary brain calcification was recently coined to refer to patients in whom neuroimaging analysis often reveals symmetrical, bilateral calcinosis in the basal ganglia, thalamus, dentate nuclei and other brain regions without a defined cause (formerly known as idiopathic brain calcification)^1^,^2^. Primary familial brain calcification, also known as Fahr’s disease, refers to cases with an autosomal dominant pattern of inheritance, ~60% of which are linked to mutations found in four genes. Two (*SLC20A2, XPR1*) have been linked to phosphate metabolism, while the other two (*PDGFB, PDGFRB*) are associated with blood–brain barrier integrity and pericyte maintenance^3^,^4^,^5^,^6^,^7^,^8^.

Few trials have been conducted to elucidate pharmacotherapy for brain calcification; the literature is limited to isolated case reports describing treatments to control tremor, headaches, mood swings, and psychotic symptoms^1^,^2^,^9^,^10^,^11^. Symptom monitoring and management is the main treatment goal.

There is an urgent need to invest in treatment options that target calcinosis *per se*, in order to halt the disease process or minimize progression. Bisphosphonates seem to be an intuitive option, considering their clinical success in various disorders that feature bone remodeling, such as Paget disease, osteoporosis, vascular calcification in kidney transplants, and bone fragmentation due to metastasis^9^,^10^,^12^. These drugs bind to hydroxyapatite crystals and thus have a very high affinity for bone^9^,^10^,^12^. Bisphosphonates are released from the bone matrix upon exposure to acid and enzymes secreted by an active osteoclast. This preserves bone matrix and increases calcium and phosphate content within bone, while decreasing bone resorption^9^,^10^,^12^. In addition, bisphosphonates cross the blood–brain barrier, making them excellent candidates for the treatment of progressive brain calcification^9^,^10^,^12^.

In 1998, experimental treatment of a familial case of primary brain calcification with a bisphosphonate (disodium etidronate) was reported^10^. The treatment produced a two-fold improvement in speech and gait, but did not affect spasticity, dystonia, or ataxia^10^. Quantitative analysis of cerebral calcification did not reveal any reduction, suggesting other possible mechanisms for this clinical improvement^10^. In 2006, the same drug was used to treat two patients with brain calcification linked to specific medical conditions: a child with seizures undergoing chemotherapy and exhibiting small spots of calcification and a patient with a cerebral hemangioma with calcifications. Both individuals were reported to have experienced improvement of seizures, headaches, and parkinsonian symptoms^9^.

Additional cases treated with bisphosphonates are necessary to ensure this class of drugs is appropriate for routine use. This paper describes 7 cases (familial and sporadic) of primary brain calcification treated with alendronate.

## Methods

Diagnostic criteria were primary brain calcification confirmed on computed tomography (CT) and exclusion of potential secondary causes, e.g., trauma or endocrine and infectious disorders, through biochemical testing and serology in blood and urine samples.

Seven patients were diagnosed with primary brain calcification (4 confirmed as familial, with specific mutations in *SLC20A2* and *PDGFB*) and started on a course of alendronate (70 mg/week). We chose alendronate due to its ease of availability, safety, and simple, once-weekly administration. All patients consented to publication of clinical data. Consent forms were obtained from patients who agreed to participate. This project was approved by the Federal University of Pernambuco IRB, registration code 09475912.8.0000.5208. All methods were in accordance with approved guidelines.

Five patients, recruited at different time points from 2012, participated in a prospective clinical protocol. Two others were retrospectively evaluated, as they had used bisphosphonates for several years for osteoporosis prior to being included in the present study.

The parameters of interest included symptoms, imaging patterns, pre- and post-treatment evaluation findings, genetic analysis, and treatment duration.

## Results

**Figure 1:**
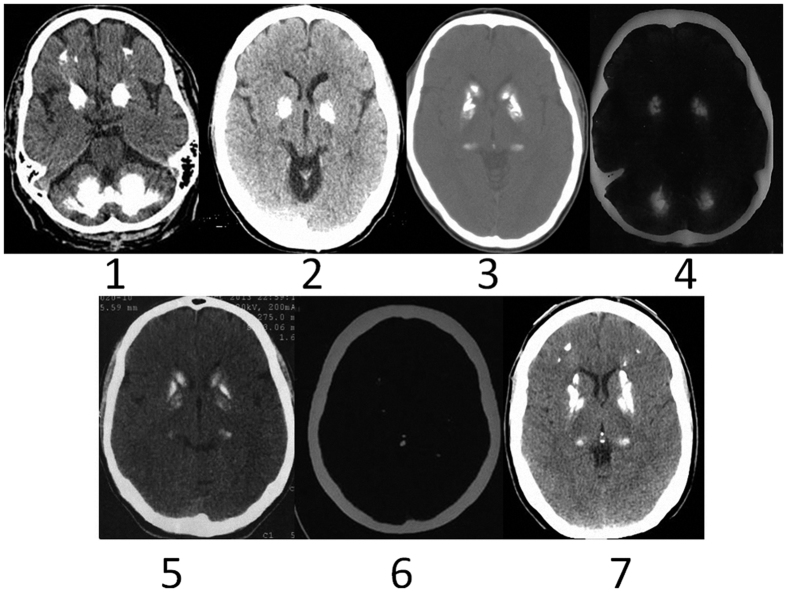
Illustrates CT images from seven patients described in the text. Above we show patients number 1, 2, 3 and 4 and below patients 5, 6 and 7.

**Figure 2:**
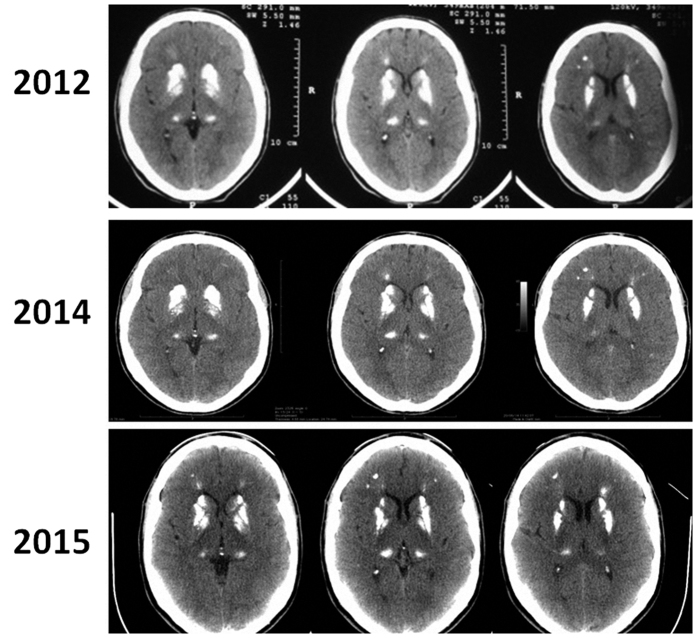
Details case 7. It is possible to see three CT separated by three years (2012, 2014 and 2015). No specific image change can be recognized, although symptoms improved after alendronate use.

Results are summarized in Table 1. Head CTs from all patients are shown in Figs 1 and 2.

Demographic characteristics, clinical profile, level of severity/impairment, genetic analysis, treatment duration, and clinical response were assessed and are presented in Table 1.

### Case Descriptions

For comparative purposes, patients will be presented in order of level of impairment, from most to least severe.

### Patient 1

Patient 1 was a 56-year-old man with a history of poliomyelitis with hypotonia and atrophy of the right lower limb. After the acute episode, he recovered limb function progressively. The patient had a normal life until age 46, when he developed slowly progressive aphasia and motor syndrome of the right lower and upper limbs. He stopped working at the age of 51 and became gradually dependent. At the time of presentation, neurological exam revealed expressive aphasia, stiffness and spasticity of the right upper and lower limbs, and lower limb atrophy. Strength, sensation, coordination, proprioception, and cranial nerves were unaffected.

The metabolic and endocrine profile (including calcium metabolism) were normal. CT scans obtained at the onset of neurological deficits showed extensive calcification of the basal ganglia, cerebellum, thalamus, and subcortical white matter. Subsequent scans confirmed progression of calcinosis. Genetic screening detected a *PDGFB* mutation (c.356 T > C). This patient received alendronate for 3 months; however, his condition was already severe, with limited movement and long periods in bed. He ultimately contracted pneumonia and died of respiratory complications.

### Patient 2

Patient 2 was a 49-year-old woman who presented with a 5-year history of progressive tremors. Cognitive examination was unremarkable; however, she had developed a depressed mood with the onset of parkinsonism. The patient received alendronate for 9 months and reported continued progression of symptoms. She continues to search for alternative treatments to improve her symptoms and slow disease progression.

### Patient 3

A 43-year-old man, one of seven children born to the same mother (described below as Patient 4), presented with rapid progression of parkinsonism. In the last 5-years, a progressive presentation of general bradykinesia, rigidity, and paresis in the right arm had developed. He had previously been an active individual with regular employment. Prior to recruitment, this patient had been on carbidopa/levodopa, which was continued throughout the duration of the present study. Genetic screening identified a *SLC20A2* mutation (c.1483 G > A)^3^, and the patient was placed on alendronate therapy.

### Patient 4

This 84-year-old woman presented with mild depression, late-stage parkinsonism, and large calcifications (10.85 cm^3^) in the basal ganglia and cerebellum. She is the mother of Patient 3 and carries the same *SLC20A2* mutation. This patient had been taking alendronate for 10 years due to a diagnosis of osteoporosis. Intriguingly, she presented with fewer symptoms than her son, despite being 41 years old older^3^.

### Patient 5

A 38-year-old male presented with sudden onset of right arm paresis followed by syncope, initially diagnosed as a stroke. Neuroimaging showed brain calcifications. While a full endocrine workup revealed no overt abnormalities, genetic analysis identified a previously reported mutation in the *SLC20A2* gene (c.1753 G > A)^3^. The patient experienced improvement in arm paresis and decreased intensity and frequency of tremors. He continues to receive alendronate.

### Patient 6

A 51-year-old female presented with a 15-year history of sporadic headaches of moderate to severe intensity, with increasing frequency over the last 6 years (current frequency: two episodes daily). She was diagnosed with osteoporosis 3 years before presentation and had been taking alendronate for the last 28 months.

### Patient 7

In this patient, a large brain calcification was discovered incidentally on head CT performed following a bicycle accident (Figs 1 and 2). The patient’s only symptoms were sporadic (weekly, mild-to-moderate headaches. Additional biochemical and hormonal tests were performed, but no abnormalities were detected. Since starting alendronate, the patient has been completely symptom-free. During 3-year neuroimaging follow-up, comparing images from 2012, 2014 and 2015 (Fig. 2), there has been no evidence of expansion.

## Discussion

Primary brain calcification is characterized by various neurological and psychiatric symptoms, and remains a diagnostic and prognostic challenge^1^,^2^,^11^. The condition is often inherited in an autosomal dominant pattern with at least two genes (*SLC20A2* and *XPR1*) associated with phosphate homeostasis^4^,^8^.

We have followed 7 patients treated with alendronate, with inconsistent results. The main finding during follow-up was stable disease. However, we observed symptomatic improvement in some cases, especially in younger patients. There was no clear change in imaging pattern during follow-up.

Treatment was well-accepted by all patients, with no reported adverse effects. An initial concern was that, for unknown reasons, alendronate treatment could trigger new symptoms; this was not the case.

Interestingly, two women (patients 4 and 6) had been on alendronate therapy for osteoporosis for several years prior to enrollment in the present study. This suggests that clinical benefit might depend on the time and duration of treatment.

Limitations include the case-series design of this study, small number of patients, absence of control group, and heterogeneous sample. Our findings may not be fully supported by the experimental data, as our results were divergent and we could not conduct any statistical analysis. Furthermore, the duration of follow-up was short. In slowly progressive diseases, such a brief follow-up period may be insufficient to allow further discussion. As clinical features were distinct, not uniform, and difficult to evaluate, no objective scales were administered. Finally, the decrease in symptom intensity (especially headache) observed during follow-up may simply correspond to an oscillating disease course.

Patients 1 and 5 presented with unilateral symptoms. Considering that calcifications are bilateral, they should generally present with symmetrical symptoms and signs. In Patient 1, unilateral symptoms might be explained by post-polio syndrome (reactivation of the disease several years after initial presentation). The unilateral symptoms of Patient 5 may eventually progress to the typical symmetrical expression.

We chose alendronate due to its availability, safety, and comfortable dosing schedule (oral administration, once a week). Etidronate probably works via a different mechanism (bulk action binding to hydroxyapatite) than the newer amino bisphosphonate alendronate (inhibition of osteoclasts). This might explain why the effects seen in our series were less dramatic than those seen in patients treated with etidronate. Thus, while alendronate has a more convenient dosing schedule and, possibly, fewer side effects, a larger clinical trial should consider the choice of bisphosphonate carefully.

To date, there is no specific treatment for primary brain calcification; the main goal is symptom management. Clinicians should make sure that the idiopathic/primary profile is accurately defined to rule out any underlying organic cause, e.g., in non-idiopathic basal ganglia calcification caused by abnormal calcium regulation, such as in primary endocrine disorders.

Bisphosphonates represent the only effective (although still anecdotal) treatment that could have wider applications in basal ganglia calcification. Prospective, controlled studies should be conducted to address the weaknesses of the present manuscript and establish a definitive analysis of bisphosphonate therapy for primary brain calcification. Furthermore, the excellent tolerability profile of alendronate in primary brain calcifications suggests that a trial in asymptomatic patients could help address the potential benefit of this strategy to control symptoms in younger patients.

## Conclusion

Biphosphonates may be applicable, safe and change the natural progression of primary brain calcifications, especially in younger patients and across prolonged periods. Nevertheless, future studies with adequate design should answer remaining questions.

## Additional Information

**How to cite this article**: Oliveira, J. R. M. and Oliveira, M. F. Primary brain calcification in patients undergoing treatment with the biphosphanate alendronate. *Sci. Rep.* **6**, 22961; doi: 10.1038/srep22961 (2016).

## Figures and Tables

**Table 1:** Summary of patients demography, main clinical profile, level of severity/impairment, genetics data, treatment duration and response.

Patient	Current age	Age of onset	Core Symptoms	Neuroimaging pattern	Clinical self accessment	Genetic analysis (Gene/mutation)	Treatment duration/follow up
1	57 (deceased)	46	Severe motor and language impairment	Large calcifications in cerebellum, thalamus, basal ganglia and white matter	Stable	PDGFB c.356 T > C	3 months
2	49	44	Moderate Parkinsonism	Large calcifications in cerebellum and basal ganglia	Gradual impairment	NA	9 months
3	48	29	Parkinsonism Depression	Large calcification in thalamus and basal ganglia	Gradual impairment	SLC20A2 c.1483G > A	24 months
4*	85	80	Depression	Large calcificiation in cerebellum and basal ganglia	Symptoms stability	SLC20A2 c.1483G > A	120 months
5	38	37	Headaches Right arm tremor	Moderate calcification in basal ganglia	Decrease in arm paresis and tremor	SLC20A2 c.1753G > A	5 months
6*	51	36	Headaches	Small calcifications in basal ganglia	Symptoms stability	NA	28 months
7	41	38	Headaches	Large calcification in basal ganglia and smaller lesion in thalamus, white matter and cerebellum	Remarkable decrease in number of episodes.	NA	20 months

For comparative reasons, the patients will be presented orderly, from the most critical (Up row) to the most functional one (bottom row), in terms of personal impairment. NA = Not available. *patients with retrospective data.
